# Bootstrap-Augmented Analysis of Non-Linear Associations Between Glucose, hsCRP, and First Myocardial Infarction in a Cardiovascular Population

**DOI:** 10.3390/ijms27042025

**Published:** 2026-02-20

**Authors:** Joanna Kostanek, Kamil Karolczak, Wiktor Kuliczkowski, Cezary Watala

**Affiliations:** 1 Department of Haemostatic Disorders, Medical University of Lodz, 6/8 Mazowiecka Street, 92-215 Lodz, Poland; kamil.karolczak@umed.lodz.pl (K.K.); cezary.watala@umed.lodz.pl (C.W.); 2 Institute for Heart Diseases, Wroclaw Medical University, 213 Borowska Street, 50-556 Wroclaw, Poland; wiktor.kuliczkowski@umed.wroc.pl

**Keywords:** myocardial infarction, cardiovascular diseases, blood biochemistry, bootstrap, retrospective study, resampling procedures

## Abstract

Myocardial infarction (MI) remains one of the most severe acute cardiac events, despite significant progress in diagnostics and therapy. Early identification of patients at risk within the broader cardiovascular disease (CVD) population is crucial for prevention and management. This study aimed to characterize the nonlinear distributions of glucose and high-sensitivity C-reactive protein (hsCRP) in patients experiencing their first MI compared with individuals hospitalized for other CVD conditions, using a bootstrap-augmented analytical approach. This retrospective study included 743 adults with confirmed CVD. Biochemical variables, including lipid profile, glucose, hsCRP, and estimated glomerular filtration rate (eGFR), were analyzed in relation to the occurrence of MI. Statistical analyses were supported by bootstrap-based validation to ensure the robustness of findings. Among the examined variables, serum glucose and hsCRP levels showed the strongest ability in discriminating MI(+) and MI(−) groups. Both variables exhibited complex, non-linear associations with the occurrence of MI, with the most pronounced differences observed in the lower and intermediate quartiles. Bootstrap-supported analyses confirmed the stability of these effects. In CVD patients, both blood glucose and hsCRP levels display non-linear relationships with the first occurrence of MI. The strongest distinctions between MI(+) and MI(−) groups were found at moderate concentrations of these variables, emphasizing the need for cautious interpretation and highlighting their role in characterizing biochemical patterns in MI(+) and MI(−) patients.

## 1. Introduction

Myocardial infarction (MI) is one of the most serious acute cardiac conditions, despite significant advancements in diagnostics and therapy. According to the universal definition of MI, its diagnosis requires confirmation of acute ischemic symptoms, characteristic changes in the EKG recording, and elevated cardiac biomarkers in peripheral blood [1].

In clinical practice, alongside biomarkers specific to myocardial damage, such as troponins, basic biochemical parameters are routinely measured. These parameters provide valuable insights into a patient’s metabolic and inflammatory status. Such tests are standard for patients with cardiovascular diseases (CVDs). Essential blood parameters, including lipid profile, glucose concentration, and high-sensitivity C-reactive protein (hsCRP), are crucial for assessing a patient’s metabolic and inflammatory condition. Dysfunctions in lipid metabolism and carbohydrate balance contribute to the progression of atherosclerosis, while elevated hsCRP levels reflect the activation of inflammatory processes associated with the destabilization of atherosclerotic plaques. These routine measurements, performed concurrently with specific cardiac biomarkers, allow for a broader understanding of the patient’s condition and supplement the diagnosis of acute coronary syndromes [2].

Elevated blood glucose levels, even in individuals without diabetes, are associated with an increased risk of CVD, including MI. A key mechanism underlying this relationship is the excessive generation of reactive oxygen species (ROS). Under normoglycemic conditions, ROS act as signaling molecules that help maintain vascular homeostasis. In contrast, chronic hyperglycemia induces excessive ROS production, resulting in oxidative stress, DNA damage, and structural as well as functional impairment of cardiovascular cells, thereby accelerating atherosclerosis [3,4]. Moreover, hyperglycemia promotes a pro-inflammatory state, commonly reflected in elevated levels of C-reactive protein (CRP). In particular, high-sensitivity CRP (hsCRP) assays enable the detection of even low-grade inflammatory activity, which is clinically relevant in patients with impaired glucose metabolism and CVD [5,6]. Importantly, CRP is not only a biomarker, but also an active mediator of endothelial dysfunction, partly through inhibition of endothelial nitric oxide synthase (eNOS) [7].

In the context of complex interdependencies among biochemical variables, such as glucose and CRP, ensuring the statistical reliability of research findings necessitates the application of advanced analytical approaches. The bootstrap procedure, first proposed by Bradley Efron, is one such method. It involves repeatedly resampling with replacement from the original dataset and calculating statistics from each sample [8]. The resulting distribution of these statistics enables the estimation of confidence intervals and hypothesis testing without requiring assumptions about the distribution of variables. In this study, the bootstrap played a crucial role in stabilizing results, particularly in situations with limited group sizes and an imbalance in their magnitudes, which is a common challenge in the analysis of retrospective clinical data.

The aim of this study was to examine the distributional characteristics of selected biochemical parameters in patients with CVD, comparing individuals with a first-ever MI to those without MI. Notably, our intention in this work was not to find or even evaluate any new diagnostic markers–potentially useful in patients with myocardial infarction, among the variables included in the analysis. The study concentrated on routinely measured clinical markers, whose accessibility and low cost make them interesting for describing biochemical patterns in cardiovascular conditions. To enhance the reliability and stability of statistical inference, bootstrap resampling procedures were applied, providing robust estimates and mitigating limitations related to sample size and variable distributions. Additionally, bootstrap-derived estimates provided more reliable effect values compared with classical estimation, offering further methodological insight into the analysis of non-linear relationships.

## 2. Results

### 2.1. Dataset Characteristics

This study draws upon a retrospective dataset comprising 743 adult individuals treated at the Department of Cardiology, Wroclaw Medical University, between 2012 and 2015. All participants had a confirmed diagnosis of CVD. Of this population, 123 patients were hospitalized due to a first-ever MI, while the remaining 620 had no documented history of MI. The dataset used for these analyses is presented in Supplementary Table S1. Key demographic, clinical, and biochemical characteristics of the study cohort (N = 743) are summarized in Table 1. Given that these variables were previously described in detail in Kostanek et al. (2025), the current presentation focuses on those most relevant to the modeling strategies applied in this analysis [9]. Clinical characteristics of the study population, including the prevalence of CVDs, comorbid conditions, and pharmacological treatment, are summarized in Supplementary Materials Table S2.

### 2.2. Preliminary Screening of Biochemical Variables via Correlation Analysis

**Figure 1:**
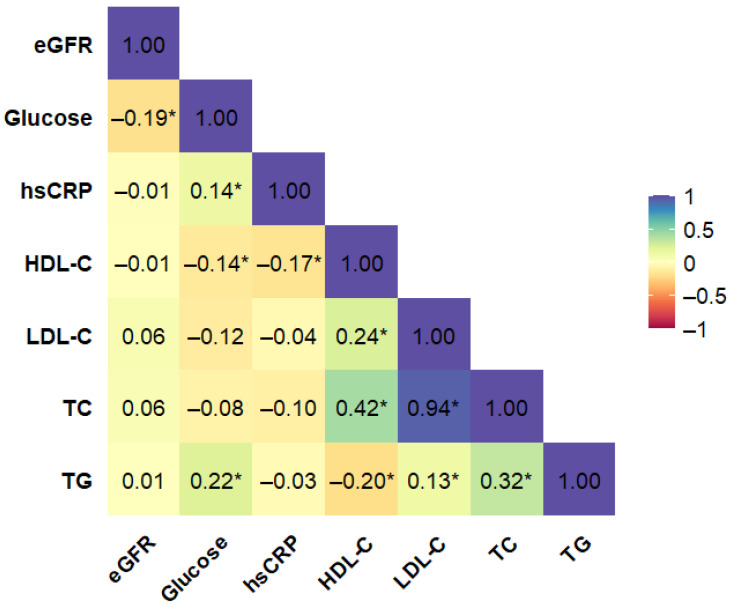
Bootstrap-boosted linear Pearson’s correlation plot of biochemical variables in a whole population of patients with cardiovascular disease (N = 743). The values represent the bootstrap-boosted linear Pearson’s correlation coefficient (10,000 iterations). Statistically significant correlations (*p* < 0.05) are marked with an asterisk (*). The analysis with the final set of biochemical variables used in subsequent modeling. Abbreviations: eGFR = estimated glomerular filtration rate, hsCRP = high-sensitivity C-reactive protein, HDL-C = high-density lipoprotein cholesterol, LDL-C = low-density lipoprotein cholesterol, TC = total cholesterol, TG = triglycerides.

To reduce multicollinearity and redundancy among biochemical variables, a correlation analysis was conducted for all included markers. The final correlation matrix, which formed the basis for subsequent modeling, is presented in Figure 1 and excludes HbA_1c_ due to the high proportion of missing data. A complementary analysis including HbA_1c_ is provided in the Supplementary Material (Figure S1), demonstrating its quite strong correlation with serum glucose level. Accordingly, HbA_1c_ was not considered in the main analyses, while its behavior was explored in complementary analyses reported in the Supplementary Material (Figure S1). The correlation analysis also revealed a strong and statistically significant association between TC and LDL-C. Therefore, to avoid potential multicollinearity, TC and LDL-C were not included simultaneously in the same regression model. All other biochemical variables showed only weak to moderate correlations and were therefore retained for statistical analysis.

### 2.3. Associations Between Selected Biochemical Markers and First-Time MI in Cardiovascular Patients

**Figure 2:**
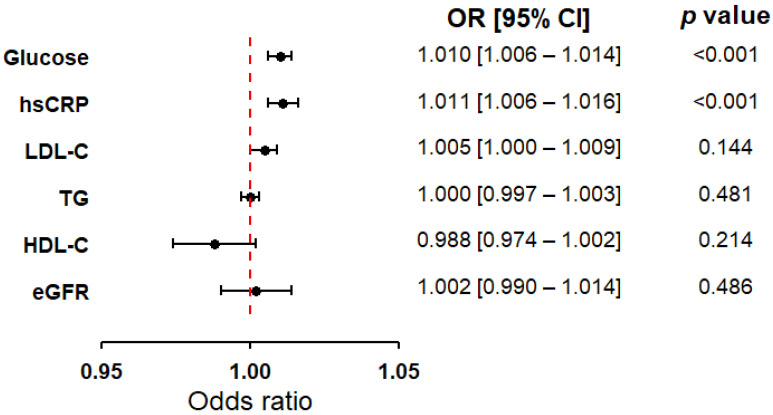
Forest plot illustrating the odds ratios (ORs) with 95% confidence intervals (CIs) for selected biochemical variables in discriminating between cardiovascular patients with current, first-time MI and those without a history of infarction. The analysis was conducted in the total study population (N = 743), with bootstrap resampling (10,000 iterations) and group balancing to 372 patients per group. Point estimates of ORs are indicated by circles, while horizontal lines represent the 95% CIs. The red dashed vertical line marks the null value (OR = 1.0), indicating no association. The logistic regression model was adjusted for age and sex. Model fit was evaluated using the Hosmer-Lemeshow test and found to be adequate (*p* > 0.05). Abbreviations: hsCRP = C-reactive protein; eGFR = estimated glomerular filtration rate; HDL-C = high-density lipoprotein cholesterol; LDL-C = low-density lipoprotein cholesterol; TG = triglycerides.

In the next step, logistic regression was applied to all biochemical markers selected after the correlation assessment. In the overall study population, only glucose levels and hsCRP concentrations showed statistically significant associations with MI, both demonstrating similar directions of effect, which justified their inclusion in subsequent models (Figure 2). The other biochemical factors did not reach statistical significance and were therefore not considered further. Given the strong correlation between TC and LDL-C, alternative models including TC instead of LDL-C are presented in Supplementary Data (Figure S2). As a complementary analysis, the regression models were additionally refitted including HbA_1c_ (Supplementary Data, Table S3) to assess the consistency of the findings. To explore potential heterogeneity, the cohort was also stratified by MI subtype: STEMI and NSTEMI (Supplementary Data, Table S4). Although selected lipid parameters reached statistical significance in certain subgroup analyses, these effects were not consistently replicated in the full cohort. Therefore, they were interpreted cautiously as exploratory findings and were not included in the subsequent main analyses. Across all steps, both classical logistic regression and bootstrap-supported models were employed to ensure the robustness of the results.

### 2.4. Non-Linear Associations Between Glucose Quartiles and First MI

To explore whether the association between serum glucose and MI status varied across the glucose range, logistic regression analyses were conducted within quartile-based glucose subgroups. Two types of models were evaluated: one including glucose alone and another combining glucose with hsCRP. Table 2 summarizes the basic characteristics of the study population stratified by glucose quartiles (Q1–Q4). Patients in Q1 were, on average, younger and exhibited a relatively balanced sex distribution. In higher quartiles, the proportion of males progressively increased, resulting in a marked sex imbalance in Q4. Rising glucose levels were accompanied by higher mean hsCRP and triglyceride concentrations, whereas HDL cholesterol and eGFR values showed a consistent decline across quartiles. The prevalence of first MI increased steadily, from 8.2% in Q1 to 28.7% in Q4.

**Figure 3:**
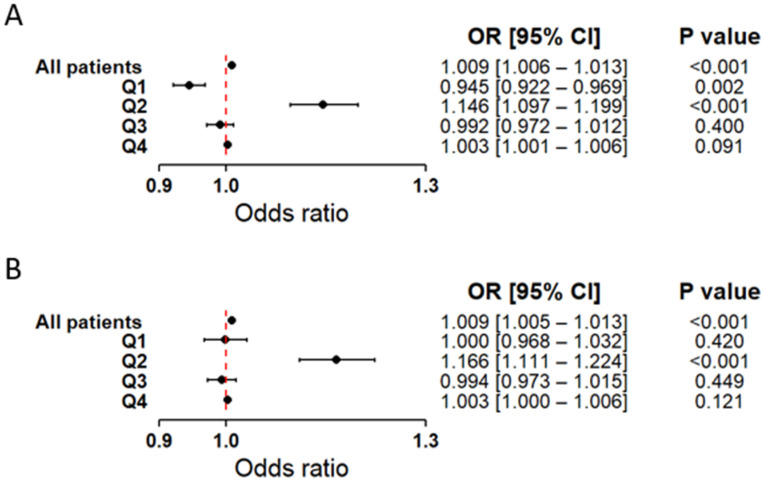
Forest plot illustrating the association between serum glucose levels and the occurrence of MI. Results are based on logistic regression models with bootstrap resampling. Odds ratios (OR) and 95% confidence intervals (CI) were calculated using 10,000 bootstrap iterations, with sample sizes adjusted to 372 patients in both the MI(+) and MI(−) groups. Circles represent point estimates of ORs, and error bars indicate 95% CIs. The dashed vertical line marks the null effect threshold (OR = 1.0). The analysis was performed in two models: (**A**) Model including glucose as the explanatory variable, adjusted for age and sex. (**B**) Model including both glucose and hsCRP as explanatory variables, also adjusted for age and sex. In both models, analyses were conducted for the overall patient cohort as well as within glucose quartile subgroups (Q1–Q4). The Hosmer-Lemeshow goodness-of-fit test yielded *p*-values greater than 0.05 for all models, indicating good model fit.

In logistic regression results non-linear relationship of glucose levels with the occurrence of a first MI became apparent (Figure 3). Interestingly, Q1 showed an inverse association with first MI, whereas Q2 was already positively and significantly related to the presence of a first MI (Figure 3A). Q3 and Q4 showed no significant dependencies. After including hsCRP in the model, the association was attenuated, with only Q2 remaining statistically significant (Figure 3B). Results from both the classical and bootstrap approaches are provided in the Supplementary Data (Table S5).

### 2.5. Quartile-Based Analysis of hsCRP and First MI

For hsCRP, quartile-based analyses were conducted using two models: one including hsCRP alone, and another combining hsCRP with glucose. The baseline clinical and biochemical characteristics of the quartile subgroups are presented in Table 3. In all quartiles except Q2, the number of men exceeded the number of women, with the greatest disproportions observed in Q1 and Q4. Increasing hsCRP concentrations were accompanied by rising serum glucose levels and progressively lower eGFR values, while the other variables showed no consistent trends. The prevalence of a first MI increased gradually across quartiles.

**Figure 4:**
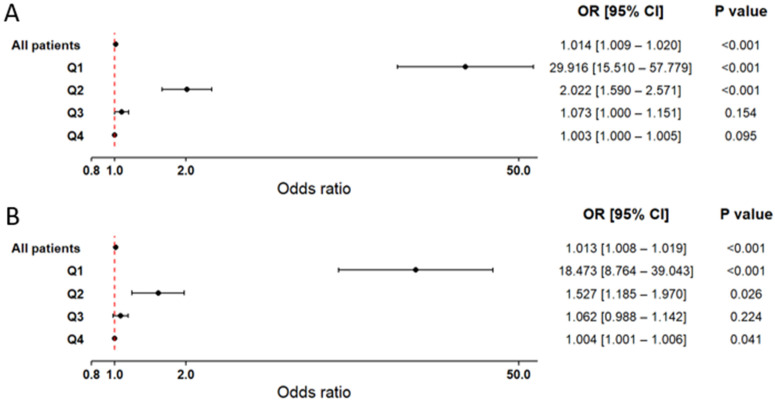
Forest plot illustrating the association between hsCRP concentrations and the occurrence of MI. Results are based on logistic regression models with bootstrap resampling. Odds ratios (OR) and 95% confidence intervals (CI) were calculated using 10,000 bootstrap iterations, with sample sizes adjusted to 372 patients in both the MI(+) and MI(−) groups. Circles represent point estimates of ORs, and error bars indicate 95% CIs. The dashed vertical line marks the null effect threshold (OR = 1.0). The analysis was performed in two models: (**A**) Model including hsCRP as the explanatory variable, adjusted for age and sex. (**B**) Model including both hsCRP and glucose as explanatory variables, also adjusted for age and sex. In both models, analyses were conducted for the overall patient cohort, as well as within hsCRP quartile subgroups (Q1–Q4). The Hosmer-Lemeshow goodness-of-fit test yielded *p*-values greater than 0.05 for all models, indicating good model fit.

Logistic regression analyses stratified by hsCRP quartiles revealed a very strong association in Q1, which remained highly significant in both the base model (Figure 4A) and after adjustment for serum glucose levels (Figure 4B). Q2 also demonstrated a statistically significant relationship, although less pronounced than in Q1. In contrast, Q3 and Q4 showed only minimal associations with the presence of a first MI that did not reach statistical significance. The observed associations proved robust, remaining consistent across classical models and bootstrap-based validation (Supplementary Table S6).

## 3. Discussion

Among the biochemical variables examined, serum glucose and hsCRP showed the most pronounced differences between patients with a first MI and those without such an event. Both variables exhibited complex, non-linear associations, with the most pronounced contrasts observed in the lower and intermediate quartiles. These effects remained robust after adjustment and bootstrap-based validation, underscoring the consistency of the findings.

The observed patterns indicate that routinely measured biochemical parameters may exhibit non-linear behavior. Group-level differences become apparent primarily when these parameters are examined within data-driven subgroups rather than through average effects. Accordingly, the findings presented here should be interpreted as a description of statistical structure within a heterogeneous clinical dataset, rather than as an assessment of diagnostic or predictive value. For glucose, individuals in Q1 were younger, exhibited the lowest prevalence of MI, and had values within the physiological range, which may account for the observed inverse relationship. Consistent with previous evidence, normoglycemia has been associated with a reduced likelihood of cardiovascular events, including MI [10]. According to our analysis, the strongest positive association between glucose levels and MI was observed in Q2. This finding is consistent with previous reports indicating that elevated admission glucose is associated with a more severe clinical course and worse in-hospital prognosis after a first MI, even among patients without diagnosed diabetes [11,12,13,14]. Moreover, increased glucose levels at hospital admission because of the first MI have been associated with a higher risk of subsequent heart failure in MI patients [15]. Elevated glucose levels may contribute to these adverse outcomes by increasing the production of reactive oxygen species (ROS). This, in turn, promotes oxidative stress and endothelial dysfunction, key processes in the pathogenesis of CVD [16]. Importantly, in the setting of AMI, these mechanisms have been directly linked to larger infarct size, enhanced inflammation, and worse prognosis [17]. Collectively, these observations emphasize the importance of early identification and tight control of glycemia during the acute phase of MI.

By contrast, the higher quartiles (Q3–Q4) of serum glucose levels or hsCRP concentrations did not reveal significant associations with MI within the cardiovascular patient cohort. This may reflect the confounding influence of age, comorbidities, and concomitant therapies. These findings suggest that moderate glucose elevations were associated with the most pronounced differences between MI(+) and MI(−) groups. In contrast, these differences became attenuated at the extremes of hyperglycemia. Nonetheless, it should be emphasized that severe hyperglycemia remains an established adverse prognostic factor after MI, being consistently linked to increased mortality and complication rates, and prior studies have demonstrated a stepwise increase in MI risk with rising glucose concentrations [18].

Our quartile-based analysis further revealed a coherent pattern of metabolic and renal alterations accompanying rising serum glucose levels. Higher glucose quartiles were associated with progressively higher triglyceride concentrations and hsCRP levels, together with lower HDL cholesterol and declining eGFR. This unfavorable clustering of metabolic disturbances is well consistent with the recognized pathophysiological links between impaired glucose homeostasis, dyslipidemia, inflammation, and renal dysfunction. Higher fasting glucose levels have been consistently linked to an adverse lipid profile, characterized by elevated triglycerides and reduced HDL-C. Moreover, the TG/HDL-C ratio is recognized as a robust marker of insulin resistance and a predictor of future diabetes, underscoring the clinical importance of monitoring these parameters for early preventive strategies [19,20]. The observed decline in eGFR across glucose quartiles also reflects the close interplay between dysglycemia and renal dysfunction. Large cohort studies indicate that a steeper decline in eGFR substantially increases cardiovascular risk in patients with diabetes. Conversely, reduced eGFR has also been associated with a higher incidence of diabetes, suggesting a bidirectional relationship between renal impairment and glucose metabolism [21,22]. Furthermore, we observed a disproportionate increase in the proportion of men in the upper quartiles, which may reflect sex-related metabolic differences. In line with previous evidence, females consistently demonstrated lower glucose levels than males [23]. These findings provide additional context for the non-linear associations between glucose levels and the occurrence of a first MI observed in our study. They suggest that even moderate glucose elevations may indicate an adverse cardiometabolic profile.

Similar to glucose levels, hsCRP concentrations demonstrated a complex, non-linear association with the occurrence of MI. The strongest difference between MI(+) and MI(−) patients was observed in the lowest quartile, where mean hsCRP values remained within the normal range. This group also showed the lowest proportion of MI cases, which may explain the pronounced contrast. Q2 likewise yielded statistically significant results, though the effect was less pronounced than in Q1. These findings are consistent with previous reports indicating that not all patients with MI present with elevated hsCRP; however, those with markedly increased levels are at higher risk of adverse outcomes and complications [24].

In our study, hsCRP showed weaker differences between MI(+) and MI(−) groups in Q3 and Q4, and these contrasts did not reach statistical significance. This may reflect the fact that markedly elevated hsCRP levels are also observed in patients without MI due to chronic inflammatory conditions or comorbidities [25]. Nevertheless, available evidence indicates that hsCRP, as a nonspecific marker of systemic inflammation, plays an important role in cardiovascular risk assessment. Among patients with MI, elevated hsCRP concentrations have been associated with an increased risk of death and recurrent cardiovascular events [26]. Importantly, hsCRP also enhances the performance of conventional risk models by identifying patients who appear to belong to a low-risk category but in fact harbor a substantially elevated susceptibility to cardiovascular events [27].

The observed heterogeneity across glucose and hsCRP quartiles may reflect complex biological variability within the studied population. This variability likely arises from overlapping metabolic and inflammatory differences across patient subgroups. Glucose homeostasis exhibits pronounced sex-related differences across the life course, which are partly modulated by sex hormones [28]. In parallel, population-based data indicate that long-term trends in CRP levels differ between women and men, suggesting sex-specific dynamics in the distribution of inflammatory markers [29]. Moreover, cardiometabolic disorders are increasing globally in both sexes; however, their prevalence and predominant pathophysiological mechanisms differ between women and men, which may further shape the distributions of metabolic and inflammatory markers observed in mixed-sex cohorts [30]. Importantly, the regression models applied in the present study were adjusted for age and sex, thereby accounting for the potential influence of these factors at the statistical level. In addition, the use of bootstrap resampling with 10,000 iterations enabled an assessment of the stability of the observed associations under varying distributions of demographic and clinical characteristics across resampled datasets. Consequently, the potential impact of residual biological variability or comorbid conditions was substantially mitigated and does not undermine the consistency of the observed findings.

In contrast to glucose and hsCRP, our study did not reveal consistent associations between the overall lipid profile and the occurrence of MI, except in the subgroup of patients with STEMI. These differences may reflect the distinct pathophysiological mechanisms underlying the two types of MI. STEMI is typically associated with sudden and complete coronary artery occlusion, often leading to extensive myocardial damage, whereas NSTEMI usually results from partial or transient obstruction, producing more limited injury [31]. Although our study did not demonstrate significant differences in lipid parameters between MI(+) and MI(−) patients, dyslipidemia remains a well-established and robust risk factor for MI [32,33]. Furthermore, reduced serum HDL-C levels in combination with elevated hsCRP have been shown to markedly increase susceptibility to coronary events among at-risk individuals [34]. Moreover, growing evidence highlights the importance of early optimization of lipid profiles as part of CVD prevention strategies, even in young individuals [35].

Given the complexity of the observed associations and the imbalance between MI(+) and MI(−) groups, reliance on conventional parametric methods alone could have resulted in biased inference. To address this, we employed bootstrap resampling, which generates empirical distributions through repeated sampling with replacement, thereby improving the precision and robustness of statistical estimates. Widely recommended in biomedical research under conditions of limited sample size or violations of parametric assumptions, this approach enabled group balancing, enhanced the stability of analyses, and provided more reliable confidence intervals and hypothesis testing [36,37]. Consequently, parametric models with greater analytical power could be applied while minimizing the risk of misleading conclusions.

Previous studies have primarily focused on the prognostic role of selected biochemical parameters in assessing cardiovascular risk and complications, including MI [38,39,40,41,42,43]. However, there is a paucity of analyses examining these markers in a cross-sectional framework that directly compares patients hospitalized with MI and those without, particularly within retrospective datasets derived from routine clinical practice. Our study addresses this gap by demonstrating that basic biochemical variables display complex, non-linear associations with the presence of MI.

This study has several limitations. First, its retrospective design precludes the establishment of causal relationships between the analyzed biochemical parameters and the occurrence of MI. Second, the analyses were conducted within a single-center cohort, which may limit the generalizability of the findings to broader populations. Finally, due to a substantial proportion of missing data, some potentially relevant variables, such as HbA1c, could not be included in the analysis. This may have influenced the overall conclusions.

## 4. Materials and Methods

### 4.1. Study Population

This study represents a subanalysis of the same patient cohort previously analyzed with respect to morphological parameters in Kostanek et al. (2025) [9]. Briefly, the cohort comprised 743 adult patients with CVD hospitalized at the Department of Cardiology, Wroclaw Medical University, between 2012 and 2015. Patients presenting with extreme deviations in key laboratory parameters—outside the reference ranges to a degree suggesting data recording or measurement error—were excluded from the analysis. The diagnosis of MI was established according to the Third Universal Definition of MI (valid until 2018). All procedures were conducted in accordance with the ethical standards of the 1975 Declaration of Helsinki for research involving human subjects. Approval was obtained from the Ethics Committee of the Wroclaw Medical University (Approval No. KB-73/2012). All participants received written information about the objectives, procedures, benefits and potential risks of the study, and provided informed consent before enrollment.

### 4.2. Sample Collection

Blood collection and processing were performed according to the same protocol as described previously in Kostanek et al., 2025 [9]. Fasting venous blood was collected from the forearm using the BD Vacutainer^®^ system (Becton Dickinson UK Ltd., Plymouth, UK) with silica-coated in line with the manufacturer’s recommendations. Samples designated for biochemical testing were processed without anticoagulants and centrifuged at 2500× *g* for 15 min at room temperature, with serum separation finalized within 4 h of collection. Whole blood for glycated hemoglobin (HbA_1c_) measurement was obtained in EDTA-containing tubes (Becton Dickinson UK Ltd., Plymouth, UK). Serum concentrations of total cholesterol (TC), triglycerides (TG), and high-density lipoprotein cholesterol (HDL) were determined using standard enzymatic colorimetric assays on the Cobas Integra 400 plus biochemical analyzer (Roche Diagnostics GmbH, Mannheim, Germany) according to the supplier’s instructions (test IDs: TC, 2nd gen—0-586; TG—0-010; HDL, 3rd gen—0-331). Low-density lipoprotein cholesterol (LDL-C) was derived using the Friedewald equation [LDL-C = TC − (HDL-C + TG/2.2)], valid for TG concentrations <4.5 mmol/L. HsCRP was quantified on the Cobas Integra^®^ 400 plus System from Roche Diagnostics GmbH (test ID: hsCRP, 0-033). Glucose levels were determined photometrically with the Cobas c system (Roche Diagnostics GmbH; Glucose HK, 3rd generation, test ID: 05 6831 6), following the manufacturer’s protocol.

### 4.3. Statistical Analysis

Statistical methods were consistent with those described previously in the morphological subanalysis of this cohort [9]. Continuous variables were expressed as medians with interquartile ranges (from lower [25%] to upper [75%] quartile), while categorical variables were summarized as percentages. The normality of the distribution was assessed using the Shapiro–Wilk test. Logistic regression was applied to estimate odds ratios (ORs) and 95% confidence intervals (CIs) for the association between biochemical variables and the presence of MI, with models adjusted for age and sex. Model calibration was assessed using the Hosmer-Lemeshow test. Parametric tests were applied in combination with a bootstrap resampling procedure to obtain robust estimates of confidence intervals and *p*-values, thereby reducing sensitivity to violations of normality assumptions. Bootstrap resampling with 10,000 iterations was used to ensure robustness. To minimize class imbalance, the study sample in both the overall and quartile-based analyses was balanced to include 372 patients with MI(+) and 372 with MI(−). All analyses were carried out using STATISTICA version 13 (TIBCO Software Inc., San Ramon, CA, USA) and R (version 4.4.0 for Windows). *p*-value < 0.05 was considered statistically significant.

## 5. Conclusions

Our study indicates that both blood glucose and hsCRP levels show nonlinear relationships with the occurrence of the first MI in the CVD patient population. By applying bootstrap resampling, we were able to obtain stable estimates of the observed distributional differences and better characterize the variability present in routinely measured biochemical parameters. The strongest distinction between the MI(+) and MI(−) groups was observed at moderate values of both variables. Comparison of classical and bootstrap-derived estimates showed that the contrasts between MI(+) and MI(−) groups were reproducible and not driven solely by sample composition. Incorporating this method provided an important methodological component, enabling a more reliable interpretation of the observed effects. The results obtained may serve as a starting point for prospective studies aimed at further exploring the biochemical patterns associated with MI and validating these observations in broader clinical cohorts.

## Figures and Tables

**Table 1:** Serum biochemistry and chosen demographic variables reported for the studied group.

Variable	Total Population n = 743	MI(−) n = 620	MI(+) n = 123	*p* Value
Age [years]	62 (53–71)	62 (52–71)	64 (57–72)	0.014
Sex				0.018 ^C^
Male, n (%)	427 (57.47)	340 (54.84)	87 (70.73)	
Female, n (%)	316 (42.53)	280 (45.16)	36 (29.27)	
Total cholesterol [mg/dL]	188 (158–220)	187 (157–220)	193.50 (160–212.50)	0.501
LDL cholesterol [mg/dL]	111 (87–139)	108 (86–140)	118 (90–136)	0.431
HDL cholesterol [mg/dL]	47 (39–56)	48 (40–57)	43 (35–50)	0.009
Triglycerides [mg/dL]	124 (94–168)	121 (93–167)	135 (104–170.50)	0.190
Glucose [mg/dL]	112 (100–139)	110 (99–133)	130 (110–167)	<0.001
HbA_1c_ [%]	5.7 (5.4–6.1)	5.6 (5.4–6.1)	5.7 (5.4–6.1)	0.386
hsCRP [mg/L]	3.47 (1.33–12.02)	2.93 (1.19–8.07)	10.69 (4.46–45.99)	<0.001
eGFR [mL/min per 1.73 m^2^]	69 (57–82)	70 (57–83)	66 (56–78)	0.211

Variables (non-adjusted) are presented as medians with interquartile ranges (from lower [25%] to upper [75%] quartile). Categorical variables were presented as percentage fractions (%), with absolute counts (n) of the whole group of investigated patients. Data for the total population (n = 743), myocardial infarction (MI(+); n = 123), and non-myocardial infarction (MI(−); n = 620) groups. Comparisons between the MI(+) and MI(−) groups were performed using unpaired Student’s *t*-test for continuous variables and Chi-square test (C) for categorical variables. All *p*-values were estimated using bootstrap resampling (10,000 iterations). Abbreviations: eGFR = estimated glomerular filtration rate, HbA_1c_ = glycated hemoglobin, hsCRP = high-sensitivity C-reactive protein, HDL cholesterol = high-density lipoprotein cholesterol, LDL cholesterol = low-density lipoprotein cholesterol.

**Table 2:** Basic characteristics of participants by quartiles of serum glucose levels.

Variable	Quartiles				
	Q1 (n = 171)	Q2 (n = 188)	Q3 (n = 183)	Q4 (n = 185)	*p* Value
Age [years]	58 (40–69)	61.5 (53–68)	63 (56–73)	65 (59–73)	<0.001
Sex					0.153 ^C^
Male, n (%)	91 (53.22)	103 (54.79)	106 (57.92)	119 (64.32)	
Female, n (%)	80 (46.78)	85 (45.21)	77 (42.08)	66 (35.68)	
Glucose [mg/dL]	93 (89–96)	105 (102–109)	122 (116–129)	168 (151–202)	<0.001
hsCRP [mg/L]	1.94 (0.72–5.85)	3.11 (1.25–6.7)	3.96 (1.56–13.3)	7.91 (2.12–25.89)	0.012
Total cholesterol [mg/dL]	187.5 (151–221)	192 (159– 221)	190.5 (157.5–225)	184.5 (157–212)	0.319
LDL cholesterol [mg/dL]	111 (85–142)	111 (88–136)	115.00 (87–144)	107 (81–130)	0.131
HDL cholesterol [mg/dL]	49 (41–57)	49 (40–59)	46.5 (38–55)	42 (36–51)	0.015
Triglycerides [mg/dL]	108.5 (87–144)	116 (90–154.5)	121 (96–168)	156 (114–203)	<0.001
eGFR [mL/min/1.73 m^2^]	74 (61–89.5)	72 (60–84)	67 (55–80)	64 (52–76)	<0.001
Myocardial infarction, n (%)					<0.001 ^C^
Yes	14 (8.19)	22 (11.70)	28 (15.30)	53 (28.65)	
No	157 (91.81)	166 (88.30)	155 (84.70)	132 (71.35)	

Variables (non-adjusted) are presented as medians with interquartile ranges (from lower [25%] to upper [75%] quartile). Categorical variables were presented as percentage fractions (%), with absolute counts (n) for the quartile groups (Q1–Q4) based on glucose values. Comparisons between quartiles were performed using one-way ANOVA for continuous variables and Chi-square test (C) for categorical variables. All *p*-values were estimated using bootstrap resampling (10,000 iterations). Abbreviations: eGFR = estimated Glomerular Filtration Rate, HDL cholesterol = high-density lipoprotein cholesterol, hsCRP = high-sensitivity C-reactive protein, LDL cholesterol = low-density lipoprotein cholesterol.

**Table 3:** Basic characteristics of participants by quartiles of hsCRP concentrations.

Variable	Quartiles				
	Q1 (n = 170)	Q2 (n = 172)	Q3 (n = 171)	Q4 (n = 172)	*p* Value
Age [years]	59 (44–69)	64 (56–73)	63 (56–71)	62 (51–71.5)	0.001
Sex					0.003 ^C^
Male, *n* (%)	104 (61.18)	82 (47.67)	95 (55.56)	115 (66.86)	
Female, *n* (%)	66 (38.82)	90 (52.33)	76 (44.44)	57 (33.14)	
Glucose [mg/dL]	106 (96–120)	110 (100–136)	114 (102–136)	127 (106–162)	<0.001
hsCRP [mg/L]	0.66 (0.43–1.04)	2.18 (1.67–2.85)	6.13 (4.36–7.92)	34.39 (17.42–76.79)	<0.001
Total cholesterol [mg/dL]	183 (156–214)	197 (164–226)	194.5 (159–224)	175 (145.5–206)	0.003
LDL cholesterol [mg/dL]	104 (84– 134)	110 (86– 142)	116 (90– 142)	106 (81– 135)	0.104
HDL cholesterol [mg/dL]	50 (42–59)	49 (41–57)	46 (37–54)	41 (33–48)	<0.001
Triglycerides [mg/dL]	116 (88–154)	134 (98–191)	128 (96–168)	124 (92–178)	0.005
eGFR [mL/min/1.73 m^2^]	75 (60– 88)	67 (58– 80)	66 (56– 79)	67 (52– 80)	<0.001
Myocardial infarction, *n* (%)					<0.001 ^C^
Yes	7 (4.12)	15 (8.72)	36 (21.05)	57 (33.14)	
No	163 (95.88)	157 (91.28)	135 (78.95)	115 (66.86)	

Variables (non-adjusted) are presented as medians with interquartile ranges (from lower [25%] to upper [75%] quartile). Categorical variables were presented as percentage fractions (%), with absolute counts (n) for the quartile groups (Q1–Q4) based on hsCRP values. Comparisons between quartiles were performed using one-way ANOVA for continuous variables and Chi-square test (C) for categorical variables. All *p*-values were estimated using bootstrap resampling (10,000 iterations). Abbreviations: eGFR = estimated Glomerular Filtration Rate, HDL cholesterol = high-density lipoprotein cholesterol, hsCRP = high-sensitivity C-reactive protein, LDL cholesterol = low-density lipoprotein cholesterol.

## Data Availability

The data supporting the reported results are provided in the Supplementary Materials.

## Supplementary Materials

The following supporting information can be downloaded at: https://www.mdpi.com/article/10.3390/ijms27042025/s1.

## Abbreviations

The following abbreviations are used in this manuscript:

AMI	Acute Myocardial Infarction
BMI	Body Mass Index
CI	Confidence Interval
CRP	C-Reactive Protein
CVD	Cardiovascular Diseases
eGFR	Estimated Glomerular Filtration Rate
eNOS	Endothelial Nitric Oxide Synthase
HbA_1c_	Glycated Hemoglobin
HDL-C	High-Density Lipoprotein Cholesterol
hsCRP	High-Sensitivity C-Reactive Protein
LDL-C	Low-Density Lipoprotein Cholesterol
MI	Myocardial Infarction
MPV	Mean Platelet Volume
NO	Nitric Oxide
NSTEMI	Non-ST-Elevation Myocardial Infarction
OR	Odds Ratio
PCT	Plateletcrit
PLCR	Platelet Large Cell Ratio
PLT	Platelets/Blood Platelets
ROS	Reactive Oxygen Species
Q1	First Quartile
Q2	Second Quartile
Q3	Third Quartile
Q4	Fourth Quartile
STEMI	ST-Elevation Myocardial Infarction
TC	Total Cholesterol
TG	Triglycerides
