# Supplementary material for: Bootstrap-Augmented Analysis of Non-Linear Associations Between Glucose, hsCRP, and First Myocardial Infarction in a Cardiovascular Population
Source: Int J Mol Sci. 2026 Feb 20;27(4):2025. doi: 10.3390/ijms27042025 (PMC12941044; doi:10.3390/ijms27042025)
Supplement: Supplementary file 1 [file ijms-27-02025-s001.zip › ijms-4135424-supplementary/Figure S1.pdf]

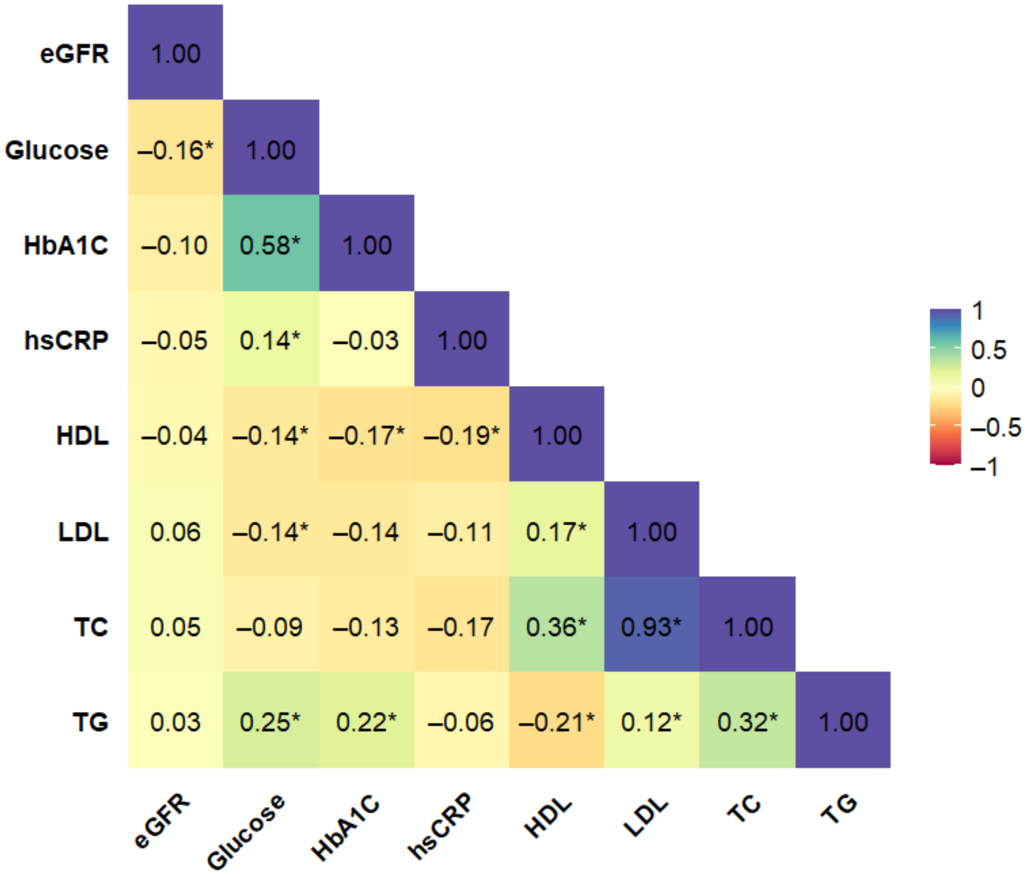

Figure S1. Bootstrap-enhanced Pearson's correlation plot of biochemical variables in a whole population of patients with cardiovascular disease (n=743). The values represent the bootstrap-boosted linear Pearson's correlation coefficient (10000 iterations). Statistically significant correlations ($P<0.05$) are marked with an asterisk (*). Abbreviations: GFR = glomerular filtration rate, Glucose = maximum glucose concentration, HbA1c = glycated hemoglobin, hsCRP = high-sensitivity C-reactive protein, HDL = high-density lipoprotein, LDL = low-density lipoprotein, TC = total cholesterol, TG = triglycerides.
