# Supplementary material for: Bootstrap-Augmented Analysis of Non-Linear Associations Between Glucose, hsCRP, and First Myocardial Infarction in a Cardiovascular Population
Source: Int J Mol Sci. 2026 Feb 20;27(4):2025. doi: 10.3390/ijms27042025 (PMC12941044; doi:10.3390/ijms27042025)
Supplement: Supplementary file 1 [file ijms-27-02025-s001.zip › ijms-4135424-supplementary/Figure S2.pdf]

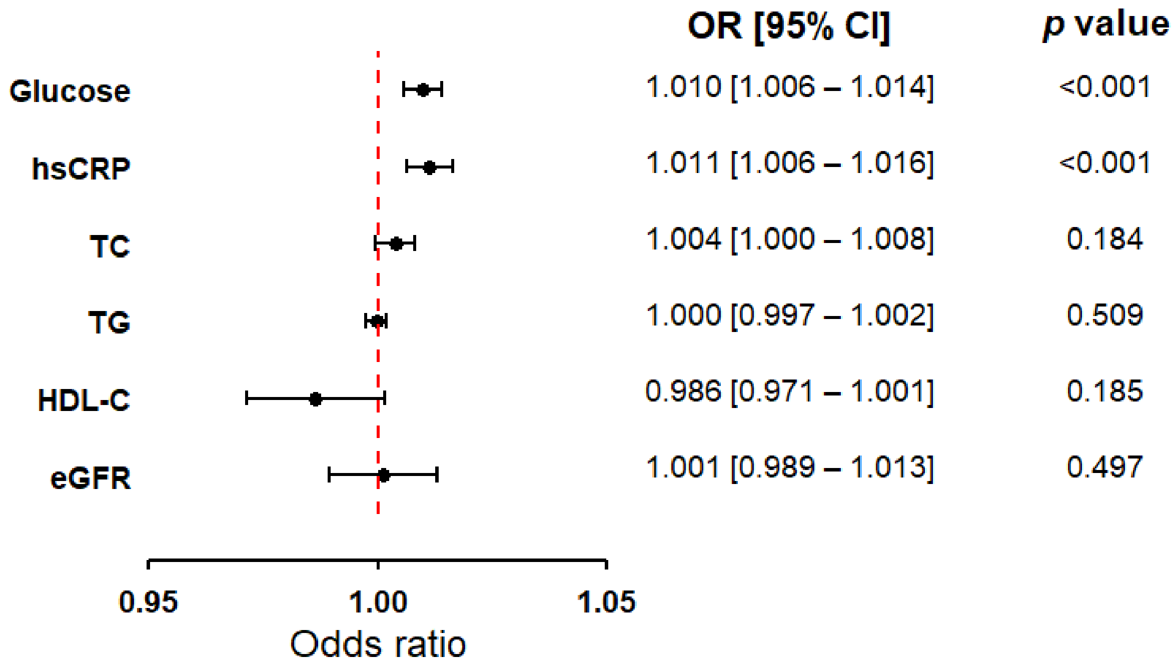

Figure S2. Forest plot illustrating the odds ratios (ORs) with 95% confidence intervals (CIs) for selected biochemical variables in discriminating between cardiovascular patients with current, first-time MI and those without a history of infarction. The analysis was conducted in the total study population (N = 743), with bootstrap resampling (10,000 iterations) and group balancing to 372 patients per group. Point estimates of ORs are indicated by circles, while horizontal lines represent the 95% CIs. The red dashed vertical line marks the null value (OR = 1.0), indicating no association. The logistic regression model was adjusted for age and sex. Model fit was evaluated using the Hosmer-Lemeshow test and found to be adequate ($P > 0.05$). Abbreviations: hsCRP = C-reactive protein; eGFR = estimated glomerular filtration rate; HDL-C = high-density lipoprotein cholesterol; TC = total cholesterol; TG = triglycerides.
