# Supplementary material for: Bootstrap-Augmented Analysis of Non-Linear Associations Between Glucose, hsCRP, and First Myocardial Infarction in a Cardiovascular Population
Source: Int J Mol Sci. 2026 Feb 20;27(4):2025. doi: 10.3390/ijms27042025 (PMC12941044; doi:10.3390/ijms27042025)
Supplement: Supplementary file 1 [file ijms-27-02025-s001.zip › ijms-4135424-supplementary/Table S2.pdf]

Table S2. Clinical characteristics of the study population, including the prevalence of cardiovascular diseases, comorbid conditions, and pharmacological treatment.

Variable	Total population	MI(+)	MI(-)	*p* Value
Myocardial infarction				
Yes	16.55 (123)			
No	83.45 (620)			
Diabetes Mellitus				0.46
Yes	21.80 (162)	24.39 (30)	21.29 (132)	
No	77.93 (579)	75.61 (93)	78.39 (486)	
Spontaneous Coronary Artery Dissection				<0.001
Yes	29.07 (216)	46.34 (57)	25.65 (159)	
No	70.12 (521)	53.66 (66)	73.39 (455)	
Congenital Heart Defect				0.05[F]
Yes	6.33 (47)	3.25 (4)	6.94 (43)	
No	93.67 (696)	96.75 (119)	93.06 (577)	
Acquired Heart Defect				0.95
Yes	53.03 (394)	52.85 (65)	53.06 (329)	
No	46.84 (348)	47.15 (58)	46.77 (290)	
Myocarditis				0.01[F]
Yes	8.21 (61)	3.25 (4)	9.19 (57)	
No	91.79 (682)	96.75 (119)	90.81 (563)	
Hypertension				0.24
Yes	68.24 (507)	73.17 (90)	67.26 (417)	
No	31.09 (231)	26.83 (33)	31.94 (198)	
Congestive Heart Failure				0.51
Yes	24.36 (181)	26.83 (33)	23.87 (148)	
No	74.16 (551)	72.36 (89)	74.52 (462)	
Arrhythmias				<0.001
Yes	46.70 (347)	24.39 (30)	51.13 (317)	
No	53.30 (396)	75.61 (93)	48.87 (303)	
Peripheral Artery Disease (Lower Limbs)				0.12
Yes	5.38 (40)	8.13 (10)	4.84 (30)	
No	92.19 (685)	87.8 (108)	93.06 (577)	
Carotid Artery Atherosclerosis				0.08
Yes	9.56 (71)	13.82 (17)	8.71 (54)	
No	86.68 (644)	82.93 (102)	87.42 (542)	
Venous Thrombosis (Lower Limbs)				0.10[F]
Yes	5.38 (40)	3.25 (4)	5.81 (36)	
No	94.48 (702)	96.75 (119)	94.03 (583)	
Pulmonary Embolism				0.08[F]
Yes	2.96 (22)	0.81 (1)	3.39 (21)	
No	96.77 (719)	98.37 (121)	96.45 (598)	
Acetylsalicylic acid				<0.001
Yes	45.76 (340)	99.19 (122)	35.16 (218)	
No	53.57 (398)	0.81 (1)	64.03 (397)	

Clopidogrel				<0.001
Yes	21.00 (156)	78.86 (97)	9.52 (59)	
No	78.20 (581)	21.14 (26)	89.52 (555)	
Ticagrelor				<0.001 [F]
Yes	1.62 (12)	9.76 (12)	0.00 (0)	
No	98.12 (729)	89.43 (110)	99.84 (619)	
Non-vitamin K oral anticoagulants				0.003
Yes	29.07 (216)	17.89 (22)	31.29 (194)	
No	70.52 (524)	82.11 (101)	68.23 (423)	
Angiotensin-converting enzyme inhibitors				<0.001
Yes	66.76 (496)	86.99 (107)	62.74 (389)	
No	33.24 (247)	13.01 (16)	37.26 (231)	
Angiotensin II receptor blockers				0.03 [F]
Yes	7.13 (53)	3.25 (4)	7.90 (49)	
No	91.79 (682)	95.12 (117)	91.13 (565)	
Beta-blockers				<0.001
Yes	77.66 (577)	95.93 (118)	74.03 (459)	
No	22.07 (164)	4.07 (5)	25.65 (159)	
Loop diuretics				0.08
Yes	16.69 (124)	11.38 (14)	17.74 (110)	
No	83.31 (619)	88.62 (109)	82.26 (510)	
Statins				<0.001 [F]
Yes	74.16 (551)	98.37 (121)	69.35 (430)	
No	25.71 (191)	1.63 (2)	30.48 (189)	
Allopurinol				0.04 [F]
Yes	6.73 (50)	3.25 (4)	7.42 (46)	
No	92.73 (689)	95.12 (117)	92.26 (572)	

Variables (non-adjusted) are presented as counts and percentages % (n) for the total cohort (N = 743) and for the MI+ and MI− groups. P-values were calculated using the Chi-square test. When the expected cell count was <5, Fisher's exact test (F) was applied.
