# Supplementary material for: Bootstrap-Augmented Analysis of Non-Linear Associations Between Glucose, hsCRP, and First Myocardial Infarction in a Cardiovascular Population
Source: Int J Mol Sci. 2026 Feb 20;27(4):2025. doi: 10.3390/ijms27042025 (PMC12941044; doi:10.3390/ijms27042025)
Supplement: Supplementary file 1 [file ijms-27-02025-s001.zip › ijms-4135424-supplementary/Table S3.pdf]

Table S3. Association between myocardial infarction and selected biochemical variables in patients with cardiovascular disease.

		Classical approach			Bootstrap approach		
		OR	95% CI	p-value	OR	95% CI	p-value
Model 1							
	Age	1.015	0.989–1.041	0.273	1.016	1.001–1.032	0.130
	Sex	1.715	0.845–3.483	0.135	1.866	1.265–2.754	0.026
	Glucose	1.011	1.005–1.018	0.001	1.013	1.009–1.018	<0.001
	HbA1c	0.820	0.541–1.241	0.347	0.793	0.630–0.998	0.140
	hsCRP	1.010	1.004–1.017	0.002	1.012	1.008–1.017	<0.001
	TC	1.008	1.001–1.016	0.031	1.009	1.004–1.013	0.003
	TG	0.997	0.991–1.002	0.240	0.996	0.993–0.999	0.061
	HDL-C	0.993	0.967–1.020	0.622	0.992	0.977–1.006	0.338
	eGFR	1.003	0.984–1.022	0.751	1.005	0.995–1.016	0.368
Model 2							
	Age	1.017	0.992–1.043	0.193	1.014	1.000–1.029	0.155
	Sex	1.597	0.819–3.113	0.170	1.673	1.158–2.417	0.053
	HbA1c	1.170	0.839–1.632	0.354	1.175	0.978–1.412	0.206
	hsCRP	1.012	1.005–1.018	<0.001	1.015	1.010–1.020	<0.001
	TC	1.007	1.000–1.014	0.055	1.007	1.003–1.011	0.010
	TG	0.998	0.993–1.003	0.388	0.998	0.995–1.000	0.204
	HDL-C	0.994	0.970–1.019	0.644	0.996	0.982–1.010	0.444
	eGFR	1.001	0.984–1.019	0.891	1.00	0.990–1.011	0.515
Model 3							
	Age	1.014	0.987–1.040	0.312	1.014	0.999–1.029	0.176
	Sex	1.690	0.836–3.419	0.144	1.815	1.233–2.672	0.032
	Glucose	1.010	1.004–1.015	<0.001	1.011	1.008–1.015	<0.001
	hsCRP	1.011	1.004–1.017	0.001	1.013	1.008–1.018	<0.001
	TC	1.009	1.001–1.016	0.027	1.009	1.005–1.013	0.002
	TG	0.997	0.991–1.002	0.214	0.996	0.993–0.999	0.037
	HDL-C	0.994	0.969–1.021	0.670	0.993	0.979–1.008	0.379
	eGFR	1.003	0.984–1.022	0.778	1.005	0.994–1.016	0.402
Model 4							
	Age	1.016	0.989–1.042	0.248	1.017	1.002–1.033	0.109
	Sex	1.700	0.835–3.460	0.143	1.842	1.246–2.723	0.032
	Glucose	1.011	1.005–1.018	<0.001	1.014	1.009–1.018	<0.001
	HbA1c	0.806	0.534–1.217	0.305	0.773	0.615–0.971	0.101
	hsCRP	1.010	1.004–1.017	0.002	1.012	1.008–1.017	<0.001
	LDL-C	1.009	1.001–1.017	0.024	1.009	1.005–1.014	0.002
	TG	0.999	0.994–1.004	0.624	0.998	0.995–1.001	0.296
	HDL-C	1.001	0.978–1.024	0.950	0.999	0.986–1.013	0.511
	eGFR	1.004	0.985–1.023	0.699	1.007	0.996–1.018	0.329
Model 5							
	Age	1.018	0.993–1.044	0.165	1.016	1.001–1.031	0.122
	Sex	1.577	0.807–3.084	0.183	1.633	1.129–2.362	0.067
	HbA1c	1.143	0.820–1.594	0.429	1.141	0.949–1.372	0.280

	hsCRP	1.012	1.005–1.018	<0.001	1.015	1.010–1.020	<0.001
	LDL-C	1.007	1.000–1.015	0.052	1.008	1.003–1.012	0.012
	TG	1.000	0.995–1.004	0.879	1.000	0.997–1.003	0.495
	HDL-C	1.000	0.978–1.022	0.997	1.002	0.990–1.015	0.487
	eGFR	1.002	0.984–1.020	0.818	1.001	0.991–1.012	0.501
Model 6							
	Age	1.014	0.988–1.041	0.284	1.015	1.000–1.030	0.161
	Sex	1.669	0.823–3.381	0.155	1.786	1.212–2.633	0.040
	Glucose	1.009	1.004–1.015	<0.001	1.011	1.008–1.015	<0.001
	hsCRP	1.011	1.004–1.017	0.001	1.013	1.008–1.018	<0.001
	LDL-C	1.009	1.001–1.017	0.021	1.010	1.005–1.014	0.001
	TG	0.998	0.993–1.004	0.549	0.998	0.995–1.001	0.214
	HDL-C	1.002	0.979–1.025	0.881	1.001	0.988–1.014	0.511
	eGFR	1.003	0.985–1.022	0.721	1.006	0.995–1.017	0.366

The results of the analysis are presented as odds ratios (OR) with confidence intervals, calculated with or without the use of the bootstrap resampling procedure (10000 iterations). Only patients with available HbA1c measurements were included. Three models are presented: model 1: TC, glucose plus HbA1c; model 2: TC, HbA1c-only; model 3: TC, glucose-only; model 4: LDL-C, glucose plus HbA1c; model 5: LDL-C, HbA1c-only; model 6: LDL-C, glucose-only. In the bootstrap approach OR values were adjusted to equal sample sizes of 372 in both the MI+ and MI− groups. The odds ratios were computed for the entire patient group (N = 743). The p-value for the Hosmer-Lemeshow test exceeded 0.05. Abbreviations: HbA1c = glycated hemoglobin, hsCRP = C-reactive protein; eGFR = estimated glomerular filtration rate; Glucose = maximum blood glucose concentration; HbA1c = glycated hemoglobin; HDL-C = high-density lipoprotein cholesterol; LDL-C = low-density lipoprotein cholesterol; TC = total cholesterol; TG = triglycerides.
