# Supplementary material for: Bootstrap-Augmented Analysis of Non-Linear Associations Between Glucose, hsCRP, and First Myocardial Infarction in a Cardiovascular Population
Source: Int J Mol Sci. 2026 Feb 20;27(4):2025. doi: 10.3390/ijms27042025 (PMC12941044; doi:10.3390/ijms27042025)
Supplement: Supplementary file 1 [file ijms-27-02025-s001.zip › ijms-4135424-supplementary/Table S4.pdf]

Table S4. Association between current myocardial infarction and selected biochemical variables in patients with cardiovascular disease: analysis in the total study population and stratified by STEMI and NSTEMI subtypes.

		Classical approach			Bootstrap approach		
		OR	95% CI	p-value	OR	95% CI	p-value
Model 1							
All patients							
	Age	1.029	1.009–1.049	0.004	1.033	1.017–1.050	0.003
	Sex	1.999	1.184–3.375	0.010	2.050	1.365–3.079	0.014
	Glucose	1.008	1.004–1.012	<0.001	1.010	1.006–1.014	<0.001
	hsCRP	1.009	1.004–1.013	<0.001	1.011	1.006–1.016	<0.001
	TC	1.005	0.999–1.011	0.081	1.004	0.9996–1.008	0.184
	TG	1.000	0.997–1.003	0.971	1.000	0.997–1.002	0.509
	HDL-C	0.985	0.965–1.006	0.153	0.986	0.971–1.001	0.185
	eGFR	0.998	0.985–1.012	0.822	1.001	0.989–1.013	0.497
STEMI							
	Age	1.020	0.989–1.052	0.217	1.022	1.005–1.038	0.065
	Sex	1.918	0.725–5.071	0.189	2.342	1.481–3.704	0.008
	Glucose	1.009	1.002–1.016	0.009	1.015	1.010–1.019	<0.001
	hsCRP	1.009	1.004–1.015	0.001	1.016	1.011–1.022	<0.001
	TC	1.013	1.002–1.023	0.018	1.012	1.007–1.018	0.002
	TG	0.998	0.992–1.004	0.562	0.997	0.993–1.000	0.155
	HDL-C	0.951	0.909–0.995	0.028	0.951	0.929–0.972	0.001
	eGFR	1.012	0.989–1.035	0.309	1.015	1.003–1.027	0.075
NSTEMI							
	Age	1.025	0.998–1.053	0.071	1.028	1.012–1.044	0.012
	Sex	1.663	0.811–3.408	0.165	1.599	1.077–2.375	0.102
	Glucose	1.006	1.000–1.012	0.038	1.009	1.004–1.013	0.003
	hsCRP	1.007	1.002–1.013	0.010	1.010	1.005–1.015	0.002
	TC	1.004	0.997–1.012	0.277	1.004	0. 9997–1.007	0.179
	TG	1.001	0.998–1.005	0.514	1.000	0.998–1.002	0.496
	HDL-C	0.987	0.960–1.016	0.377	0.988	0.975–1.002	0.195
	eGFR	0.996	0.977–1.015	0.671	0.998	0.987–1.010	0.491
Model 2							
All patients							
	Age	1.029	1.009–1.049	0.004	1.033	1.017–1.050	0.003
	Sex	1.938	1.142–3.292	0.014	1.964	1.302–2.963	0.023
	Glucose	1.008	1.004–1.013	<0.001	1.010	1.006–1.014	<0.001
	hsCRP	1.009	1.004–1.013	<0.001	1.011	1.006–1.016	<0.001
	LDL-C	1.006	1.000–1.011	0.057	1.005	1.000–1.009	0.144
	TG	1.000	0.997–1.004	0.851	1.000	0.997–1.003	0.481
	HDL-C	0.988	0.969–1.006	0.196	0.988	0.974–1.002	0.214
	eGFR	0.999	0.985–1.013	0.867	1.002	0.990–1.014	0.486
STEMI							
	Age	1.020	0.989–1.052	0.206	1.023	1.006–1.039	0.056
	Sex	1.905	0.715–5.079	0.198	2.339	1.476–3.709	0.008
	Glucose	1.009	1.002–1.016	0.011	1.014	1.009–1.019	<0.001

	hsCRP	1.010	1.004–1.015	<0.001	1.016	1.011–1.022	<0.001
	LDL-C	1.012	1.002–1.023	0.024	1.012	1.006–1.018	0.003
	TG	1.002	0.996–1.008	0.602	1.000	0.997–1.004	0.504
	HDL-C	0.964	0.926–1.003	0.070	0.963	0.944–0.983	0.006
	eGFR	1.012	0.989–1.036	0.308	1.015	1.003–1.028	0.081
NSTEMI							
	Age	1.025	0.998–1.052	0.072	1.028	1.011–1.044	0.014
	Sex	1.570	0.755–3.264	0.227	1.500	1.005–2.239	0.163
	Glucose	1.007	1.001–1.013	0.026	1.010	1.006–1.015	0.001
	hsCRP	1.007	1.001–1.013	0.013	1.010	1.005–1.015	0.003
	LDL-C	1.006	0.998–1.014	0.121	1.006	1.002–1.011	0.045
	TG	1.000	0.995–1.005	0.929	0.999	0.995–1.002	0.385
	HDL-C	0.986	0.961–1.013	0.307	0.987	0.974–1.000	0.143
	eGFR	0.996	0.977–1.016	0.723	1.000	0.988–1.013	0.501

The results of the analysis are presented as odds ratios (OR) with confidence intervals, calculated with or without the use of the bootstrap resampling procedure (10000 iterations). Model 1: TC; model: LDL-C. In the bootstrap approach OR values were adjusted to equal sample sizes of 372 in both the MI+ and MI− groups. The odds ratios were computed for the entire patient group (N = 743). The p-value for the Hosmer-Lemeshow test exceeded 0.05. Abbreviations: hsCRP = C-reactive protein; eGFR = estimated glomerular filtration rate; Glucose = maximum blood glucose concentration; HDL-C = high-density lipoprotein cholesterol; LDL-C: low-density lipoprotein cholesterol; TC = total cholesterol; TG = triglycerides.
