# Supplementary material for: Bootstrap-Augmented Analysis of Non-Linear Associations Between Glucose, hsCRP, and First Myocardial Infarction in a Cardiovascular Population
Source: Int J Mol Sci. 2026 Feb 20;27(4):2025. doi: 10.3390/ijms27042025 (PMC12941044; doi:10.3390/ijms27042025)
Supplement: Supplementary file 1 [file ijms-27-02025-s001.zip › ijms-4135424-supplementary/Table S5.pdf]

Table S5 Association between myocardial infarction and glucose and hsCRP in cardiovascular patients with varying glucose levels.

Model I		Classical approach			Bootstrap approach		
		OR	95% CI	p-value	OR	95% CI	p-value
Q1 (N=171)							
	Age	1.048	1.010–1.088	0.013	1.056	1.044–1.068	<0.001
	Sex	4.894	1.245–19.247	0.023	6.255	4.275–9.154	<0.001
	Glucose	0.943	0.865–1.028	0.179	0.945	0.922–0.969	0.002
Q2 (N=188)							
	Age	1.026	0.988–1.064	0.182	1.038	1.024–1.054	<0.001
	Sex	1.544	0.599–3.983	0.369	1.485	1.084–2.035	0.086
	Glucose	1.141	0.998–1.305	0.054	1.146	1.097–1.199	<0.001
Q3 (N=183)							
	Age	1.032	1.0001–1.066	0.049	1.036	1.024–1.049	<0.001
	Sex	2.269	0.925–5.566	0.074	2.270	1.647–3.128	<0.001
	Glucose	0.986	0.933–1.042	0.619	0.992	0.972–1.012	0.400
Q4 (N=185)							
	Age	0.995	0.966–1.024	0.716	0.995	0.982–1.009	0.433
	Sex	2.015	0.978–4.154	0.058	2.094	1.518–2.889	0.001
	Glucose	1.003	0.997–1.010	0.306	1.003	1.001–1.006	0.091
Model II		Classical approach			Bootstrap approach		
		OR	95% CI	p-value	OR	95% CI	p-value
Q1 (N=171)							
	Age	1.045	1.003–1.089	0.036	1.044	1.032–1.056	<0.001
	Sex	3.610	0.804–16.222	0.094	6.205	3.977–9.689	<0.001
	Glucose	0.968	0.872–1.074	0.534	0.9995	0.968–1.032	0.420
	hsCRP	1.015	1.003–1.027	0.013	1.027	1.018–1.036	<0.001
Q2 (N=188)							
	Age	1.031	0.990–1.074	0.143	1.047	1.030–1.064	<0.001
	Sex	1.402	0.509–3.861	0.514	1.345	0.956–1.893	0.216
	Glucose	1.146	0.990–1.327	0.068	1.166	1.111–1.224	<0.001
	hsCRP	1.004	0.993–1.016	0.461	1.011	1.004–1.017	0.028
Q3 (N=183)							
	Age	1.035	1.002–1.068	0.035	1.043	1.030–1.057	<0.001
	Sex	1.891	0.756–4.733	0.173	1.954	1.392–2.744	0.007
	Glucose	0.984	0.928–1.043	0.588	0.994	0.973–1.015	0.449
	hsCRP	1.009	1.001–1.017	0.030	1.014	1.009–1.018	<0.001
Q4 (N=185)							
	Age	0.992	0.963–1.022	0.598	0.991	0.977–1.005	0.304
	Sex	1.956	0.926–4.132	0.079	2.031	1.451–2.843	0.004
	Glucose	1.002	0.996–1.009	0.460	1.003	1.000–1.006	0.121
	hsCRP	1.010	1.003–1.017	0.006	1.012	1.008–1.016	<0.001

The results of the analysis are presented as odds ratios (OR) with confidence intervals, calculated with or without the use of the bootstrap resampling procedure (10000 iterations). OR values were adjusted to equal sample sizes of 372 in both the MI+ and MI− groups. The analysis was performed across three distinct models to investigate the influence of selected variables on the myocardial infarction. Model I examined the effects of age, sex, and glucose. Model II, in addition to age and sex, included glucose, and hsCRP. The odds ratios were computed for the entire patient group, stratified based on quartiles of glucose values. In all models, the p-values for the Hosmer-Lemeshow test were greater than 0.05. Abbreviations: hsCRP = high-sensitivity C-reactive protein
