# Supplementary material for: Bootstrap-Augmented Analysis of Non-Linear Associations Between Glucose, hsCRP, and First Myocardial Infarction in a Cardiovascular Population
Source: Int J Mol Sci. 2026 Feb 20;27(4):2025. doi: 10.3390/ijms27042025 (PMC12941044; doi:10.3390/ijms27042025)
Supplement: Supplementary file 1 [file ijms-27-02025-s001.zip › ijms-4135424-supplementary/Table S6.pdf]

Table S6 Association between myocardial infarction and glucose and hsCRP in cardiovascular patients with varying hsCRP levels.

Model I		Classical approach			Bootstrap approach		
		OR	95% CI	p-value	OR	95% CI	p-value
Q1 (N=170)							
	Age	1.011	0.960–1.065	0.685	1.032	1.018–1.045	<0.001
	Sex	4.449	0.515–38.436	0.175	12.180	7.346–20.210	<0.001
	hsCRP	7.249	0.668–78.694	0.104	29.916	15.510–57.779	<0.001
Q2 (N=172)							
	Age	1.011	0.971–1.053	0.587	1.014	1.001–1.027	0.108
	Sex	1.337	0.456–3.915	0.597	1.434	1.062–1.936	0.105
	hsCRP	1.852	0.821–4.178	0.138	2.022	1.590–2.571	<0.001
Q3 (N=171)							
	Age	1.042	1.006–1.079	0.020	1.041	1.027–1.055	<0.001
	Sex	3.404	1.451–7.983	0.005	3.409	2.458–4.727	<0.001
	hsCRP	1.067	0.906–1.256	0.439	1.073	1.0004–1.151	0.154
Q4 (N=172)							
	Age	1.028	1.006–1.050	0.013	1.031	1.020–1.042	<0.001
	Sex	1.294	0.641–2.613	0.472	1.345	0.975–1.856	0.201
	hsCRP	1.003	0.998–1.008	0.264	1.003	1.0004–1.005	0.095
Model II		Classical approach			Bootstrap approach		
		OR	95% CI	p-value	OR	95% CI	p-value
Q1 (N=170)							
	Age	1.004	0.947–1.064	0.899	1.040	1.023–1.057	<0.001
	Sex	4.847	0.451–52.117	0.193	11.125	6.210–19.965	<0.001
	Glucose	1.018	1.001–1.036	0.044	1.031	1.024–1.038	<0.001
	hsCRP	4.434	0.333–58.988	0.259	18.473	8.764–39.043	<0.001
Q2 (N=172)							
	Age	1.005	0.959–1.054	0.831	1.011	0.997–1.025	0.225
	Sex	1.647	0.493–5.500	0.417	1.505	1.083–2.090	0.097
	Glucose	1.012	1.002–1.021	0.018	1.011	1.007–1.014	<0.001
	hsCRP	1.487	0.599–3.693	0.392	1.527	1.185–1.970	0.026
Q3 (N=171)							
	Age	1.040	1.004–1.077	0.027	1.038	1.024–1.053	<0.001
	Sex	3.174	1.325–7.600	0.010	3.189	2.280–4.461	<0.001
	Glucose	1.003	0.996–1.010	0.401	1.003	1.0000–1.007	0.143
	hsCRP	1.056	0.892–1.251	0.527	1.062	0.988–1.142	0.224
Q4 (N=172)							
	Age	1.030	1.007–1.055	0.011	1.034	1.023–1.046	<0.001
	Sex	1.349	0.655–2.779	0.416	1.446	1.037–2.016	0.132
	Glucose	1.003	0.997–1.010	0.345	1.004	1.0005–1.007	0.112
	hsCRP	1.003	0.998–1.008	0.188	1.004	1.001–1.006	0.041

The results of the analysis are presented as odds ratios (OR) with confidence intervals, calculated with or without the use of the bootstrap resampling procedure (10000 iterations). OR values were adjusted to equal sample sizes of 372 in both the MI+ and MI− groups. The analysis was performed across three distinct models to investigate the influence of selected variables on the myocardial infarction. Model I examined the effects of age, sex, and hsCRP. Model II, in addition to age and sex, included glucose, and hsCRP. The odds ratios were computed for the entire patient group, stratified based on quartiles of hsCRP values. In all models, the p-values for the Hosmer-Lemeshow test were greater than 0.05. Abbreviations: hsCRP = high-sensitivity C-reactive protein
